# A Life-Saving Thought

**Published:** 1855-06

**Authors:** 


### A LIFE-SAVING THOUGHT.

An amount of sickness, suffering and death will be saved to multitudes during any spring and summer, if the suggestions which I am about to make were attended to.

Children eat for three objects:

1. To keep them warm,
2. To supply the wastes of the system,
3. To afford materials for growth.

Hence, children who are in health, are always hungry, are always eating; we can well remember the happy time when we could eat apples all day, and melons and grapes and gingerbread and candies, besides the regular meals of morning, noon and night. But in mature life, the experience of each will tell him how changed; the reason is, one object of eating has ceased to exist—we grow no longer, and nature, with her watchful instinct, steps in, and moderates the appetite; for if we ate as when we were children, very few would survive a third of a century.

The objects, then, for which men eat are two only: first, to keep them warm; second, to supply the wastes of the system—and whatever is eaten beyond what is necessary for these two things, engenders disease in every body, everywhere, and under all circumstances, and never fails, no more than fails the rising of the daily sun, for nature's laws are constant as the flow of time.

No man works as hard in summer as in winter, consequently the wastes of the system are less; therefore a less amount of food is wanted in summer than in winter. The supply must be regulated by the demand.

Again, we eat to keep us warm. Some articles of food have ten times more fuel than nutriment. [See *Bronchitis and Kindred Diseases*, 8th ed., p. 290.] It must therefore be apparent, that we do not require as much food in summer as in winter for this reason also, that there is not the same demand for heat, and kind nature, ever watchful, steps in again and takes away our appetite as soon as the warm weather begins. All of us are sensible of a diminution of appetite even in early spring. But forgetting the natural reasons for it, we begin to think we are not well. and either by tempting the appetite, or taking tonics, or "*forcing*" food, crowd the system with more aliment than the body requires. For a while, the bodily powers, with the excess of winter vigor, are able to work up this extra supply, and convert it into blood, but there is no use for it all, it is not called for, and it accumulates in the body, stagnates, or, in medical phrase, causes "congestions." Congestion in the brain, causing us to feel dull and heavy and stupid and sleepy: congestion in the stomach causes loss of appetite: congestion in the liver gives rise to nausea, sick headache, diarrhœas, dysenteries, and the whole catalogue of fevers.

The brute creation, obeying their instinct, are not troubled with *summer complaints*, and the thousand ills which affect and destroy men. But we overpower our instincts, and making ourselves the slaves of appetite, contrary to reason, perish in multitudes. Investigations have shown, that *we require in mid-summer, near one-half less food than in mid-winter.* I throw this great practical truth before the people, and for the present, leave it.

